# Early Detection of Radiation Pneumonitis on Cone-Beam CT Images During a Course of Radiotherapy: A Case Series Report

**DOI:** 10.7759/cureus.38275

**Published:** 2023-04-29

**Authors:** Rieko Azumi, Maki Soyama, Mari Saito

**Affiliations:** 1 Radiology, National Hospital Organization (NHO) Nishiniigata Chuo Hospital, Niigata, JPN; 2 Radiology, Niigata Diagnostic Imaging Center, Niigata, JPN

**Keywords:** lung image, radiotherapy (rt), cone-beam computed tomography (cbct), early detection, radiation pneumonitis

## Abstract

Background and aim

Radiation pneumonitis (RP) is a critical pulmonary toxicity following dose delivery to the lung, and it is usually diagnosed after radiotherapy courses are completed. Because RP may result in a lethal complication, a practical method for detecting early-phase RP is awaited. In this article, we describe our experience through a variety of clinical cases and discuss treatment decisions and lessons we have made and learned.

Materials and methods

A daily cone-beam computed tomography (CBCT) scan was employed with a lung window setting to detect the early-phase RP during treatment courses. For the past five years, thirty patients were diagnosed with RP, and eight patients were detected during radiotherapy courses on the CBCT images. Our best efforts were made in detecting early ground-glass opacity and early RP on CBCT images prior to symptoms. The eight cases were described in more detail with CBCT or CT images.

Results and discussion

Initially, RP was detected at 50 Gy or greater. However, more careful CBCT observation resulted in earlier detection at around 40 Gy. Then, a new problem arose whether the treatment should be terminated. It was reported that early RP development was associated with higher-grade complications, and therefore it is preferable to terminate radiotherapy once we detect even early-phase RP. However, termination in the middle of the treatment course may significantly reduce the therapeutic effect. In our experience, patients with favorable clinical status may continue to receive radiotherapy with careful observation of lung parenchyma on CBCT images and clinical data, such as Krebs Von den Lungen-6 (KL-6) and C-reactive protein (CRP).

Conclusion

We have shown that early detection of RP may be feasible during radiotherapy courses by daily monitoring of CBCT lung images. Further studies are awaited to proceed.

## Introduction

Radiation pneumonitis (RP) is one of the most clinically challenging toxicities that may happen following lung radiation [1,2]. In the past, many oncologists analyzed this risk, leading to three major findings. First, the volume having at least 20 Gy (V20) increased RP risks. For example, it was reported that V20>40% in 3D treatment planning was the only risk factor in the multivariate analysis [3], and V20 was the only risk factor in the univariate analysis for concurrent chemoradiation [4]. It was also shown that V20>20% was the highest risk factor in the univariate and multivariate analyses [5], whereas V20>26% was a high-risk factor in the univariate and multivariate analyses for concurrent chemoradiation [6]. Second, It was reported that pre-existing interstitial pneumonia or interstitial pulmonary fibrosis resulted in severe RP with conventional fractionation [6-8] and hypofractionated stereotactic radiotherapy [9,10]. If additional chemotherapy was further employed, more severe RP occurred [8]. Third, the early appearance of symptoms developed more severe complications with conventional fractionation [7,8,11] and stereotactic radiotherapy [12]. For example, it was reported that, out of 106 patients, mean times to develop RP in grades 1, 2, 3, and 5 were 4.6, 2.3, 0.3, and 0.4 months, respectively [7]. In more detail, the grade 5 RP happened in five out of the 106 patients within one month after radiotherapy, while two out of the five grade 5 incidences occurred during the course of radiotherapy [7].

RP may be confirmed by a CT scan after significant symptoms appear during or post-radiotherapy. If a patient remains asymptomatic, RP may be confirmed by blood tests such as Krebs Von den Lungen-6 (KL-6) [13] or C-reactive protein (CRP) and subsequent CT scans during follow-up visits. Because RP could be a lethal complication, early detection may be desirable during a course of radiotherapy; however, no standard methods have been developed yet for the detection of early RP. In our hospital, early-phase RP in some patients has been observed on daily cone-beam CT (CBCT) scans during radiotherapy courses using an Elekta Synergy® linac (Elekta AB, Stockholm, Sweden). The purpose of this retrospective study was to report our experience through a variety of clinical cases, including CBCT images that suggested early-phase RP.

## Materials and methods

Between January 2016 and January 2021, 161 patients received a dose to their lung or mediastinum, and 30 patients were diagnosed with RP, excluding those who were diagnosed with pulmonary fibrosis on CT scans exceeding one year after completion of the treatment. Twenty-two out of the 30 patients were diagnosed with grade 1 or higher RP after the radiotherapy was completed. The remaining eight patients were diagnosed with grade 2 or higher RP on daily CBCT images with an imaging dose of 16 mGy during their radiotherapy courses, as summarized, where the toxicities were graded according to Common Terminology Criteria for Adverse Events (CTCAE) version 5 [14], and the lung V20 (volume receiving at least 20 Gy) was calculated at the time of treatment planning. The pretreatment pulmonary fibrosis status and its score were also indicated [6]. Written informed consent was obtained from the patients, and the study was approved by NHO Nishiniigata Chuo Hospital Ethics Committee (#2301).

For each of the eight patients mentioned above who were diagnosed with grade 2 or higher RP during their radiotherapy courses, RP was detected by the presence of ground-glass opacity (GGO) alone or consolidation with GGO on daily CBCT images. These patients further underwent high-resolution CT scan on the same day for reconfirmation. The planned target dose was 60 Gy in 30 fractions, excepting case 6, with a prescribed dose of 54 Gy in 30 fractions. Our principle was that dose delivery was terminated when RP was detected. However, in case 2 only, dose delivery was continued to a full dose of 60 Gy due to a strong desire from the patient. Case 3 had pre-existing obstructive pneumonia and slight pretreatment pulmonary fibrosis, resulting in grade 3 RP during the course of radiotherapy, thus leading to immediate termination of the treatment. In case 6, RP was arguably recognized at around 36 Gy and more firmly confirmed at 43.2 Gy. Cases 5 through 7 had asymptomatic grade 2 RP, which were confirmed by increased serum levels of KL-6 and CRP. These patients were treated with oral administration of prednisolone. For grade 3 patients (cases 3 and 4), intravenous pulse prednisolone was administered under hospitalization.

Firstly, cases 1, 2, 3, and 6 are described in more detail. Secondly, the remaining cases, 4, 5, 7, and 8, are briefly reviewed. Then, the 22 cases who were diagnosed with RP after completing radiotherapy are also summarized.

## Results

Table 1 summarizes the characteristics of the eight patients who suffered from RP during their radiotherapy courses. Planned target doses were 60 Gy in 30 fractions except for case 6, with a prescription of 54 Gy in 30 fractions. The cases are listed in chronological order of the radiotherapy.

**Table 1:** Characteristics of the eight patients who suffered from radiation pneumonitis during their radiotherapy courses This table includes pre- or concurrent chemotherapy status, doses at the RP detection, pretreatment pulmonary fibrosis status with its score in parentheses, pretreatment COPD stages, RP grades according to CTCAE v5, symptoms, lung V20, prednisolone administration for RP and the resulting outcomes. + slightly; ± barely; * hospitalized RP - radiation pneumonitis; COPD - chronic obstructive pulmonary disease; CTCAE - Common Terminology Criteria for Adverse Events; V20 - volume receiving at least 20 Gy

Case	Age, gender	Site	Pre- or concurrent chemotherapy	Dose at RP detection	Pretreatment pulmonary fibrosis (score)	Pretreatment COPD stage	RP grade	Symptom	Lung V20 (%)	Prednisolone administration	RP outcomes
												1	81M	lung + anterior chest wall	-	54 Gy	- (0)	-	G2	fever	32	oral	relief
												2	81M	lung + spine	-	50 Gy	- (0)	-	G2	cough	10	oral	relief
												3	83M	lung	-	54 Gy	+ (1)	II	G3	mild fever	13	intravenous pulse*	relief
4	81M	lung + mediastinum	+	58 Gy	± (1)	-	G3	mild fever	20	intravenous pulse*	relief
5	80F	lung + mediastinum	-	56 Gy	± (1)	-	G2	-	24	oral	relief
6	53M	lung + mediastinum	+	43.2 Gy	- (0)	-	G2	-	19	oral	relief
7	88F	lung + mediastinum	-	42 Gy	- (0)	I	G2	-	14	oral	relief
8	79M	lung + mediastinum	-	50 Gy	- (0)	-	G2	mild fever	15	oral	relief

Case 1

**Figure 1:**
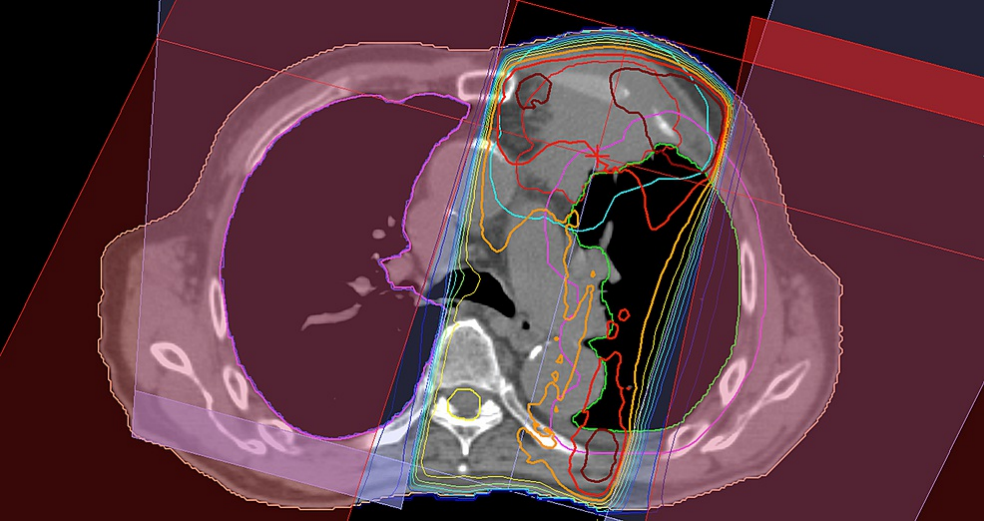
A treatment planning result A clinical target volume (CTV) and a planning target volume (PTV) were delineated in thin red and sky-blue lines, respectively. Isodose contours were drawn in thick lines. Immediately after delivering 40 Gy, another boost plan to the shrinking tumor was created to deliver an additional 20 Gy, thereby sparing the spinal cord.

**Figure 2:**
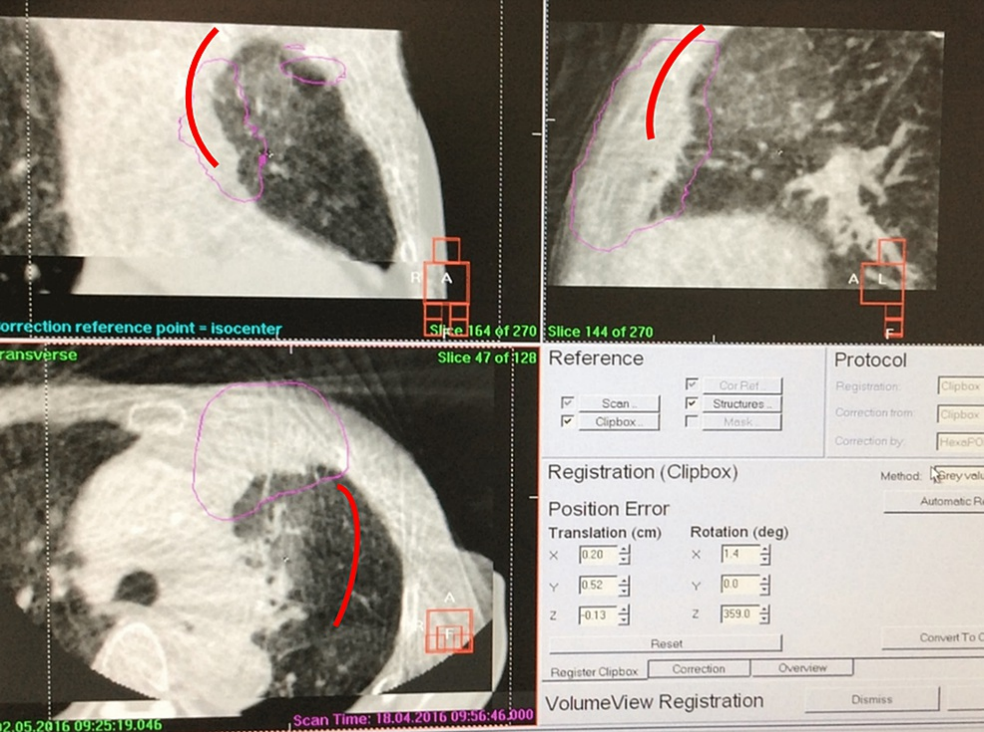
CBCT images in three orthogonal planes with a lung window setting RP is suggested by the red lines. On each of the previous treatment days, a mediastinal window setting was employed for tumor registration, and the window setting was changed to lung because the patient showed RP symptoms. Our retrospective investigation revealed that we could have observed RP three days earlier if the lung window setting had been employed. CBCT - cone-beam computed tomography; RP - radiation pneumonitis

**Figure 3:**
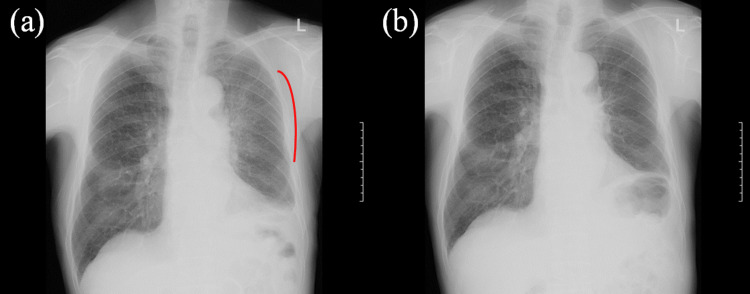
X-ray photographs X-ray photograph (a) with a clear consolidation shadow marked with a red line in the left upper lung field, which was acquired on the day the CBCT images suggested RP. Then, oral prednisolone administration was started. Another X-ray photograph (b) was acquired a week later, the shadow being undetected. CBCT - cone-beam computed tomography; RP - radiation pneumonitis

CT scan revealed lung cancer with anterior chest wall invasion. Figures 1-2 show a treatment plan result and daily CBCT images in three orthogonal planes with a lung window setting, respectively. Figures 3a-b demonstrate changes in X-ray photographs a week before and after oral prednisolone administration. In this case, RP was found on the CBCT images at 54 Gy, and the radiotherapy was therefore terminated with a dose of 6 Gy remaining undelivered.

Case 2

**Figure 4:**
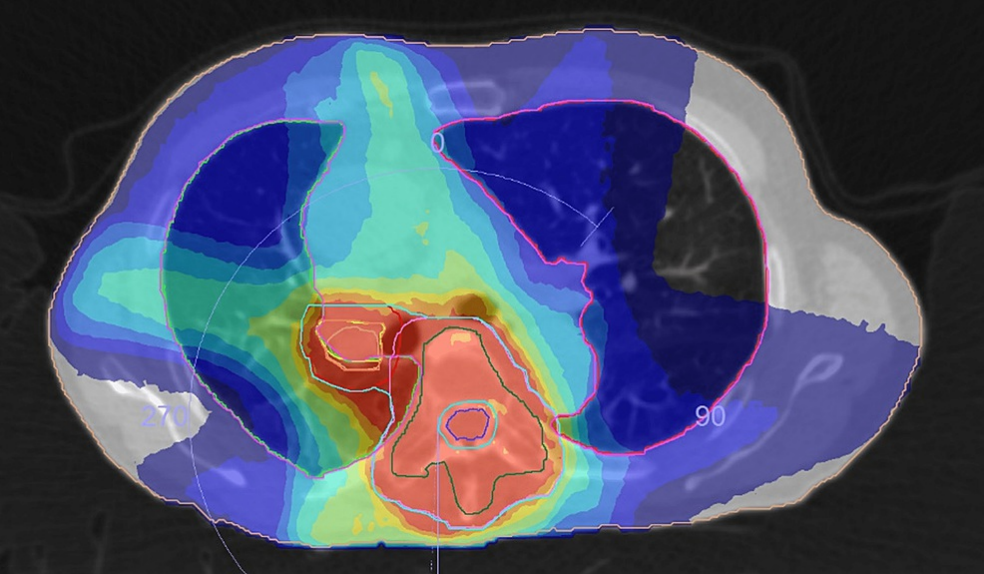
A treatment planning result PTV was delineated with a thin sky-blue line that covered the primary lung cancer and the bone metastases, the figure being further overlaid with a dose color wash. Immediately after delivering 40 Gy, another boost plan only to the shrinking lung cancer was created to deliver an additional 20 Gy, thereby sparing the spinal cord. PTV - planning target volume

**Figure 5:**
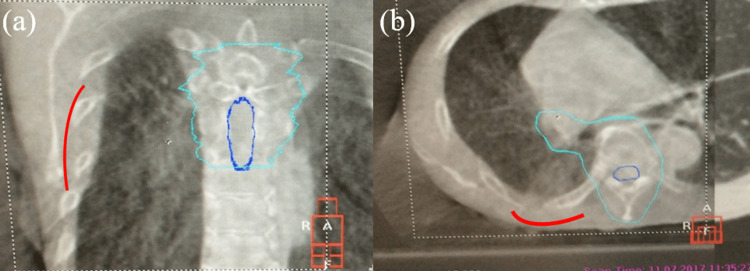
CBCT images displayed in (a) coronal and (b) axial planes RP was suggested by the red lines. Sky-blue and blue lines show the PTV and spinal cord contours, respectively. RP - radiation pneumonitis; PTV - planning target volume

**Figure 6:**
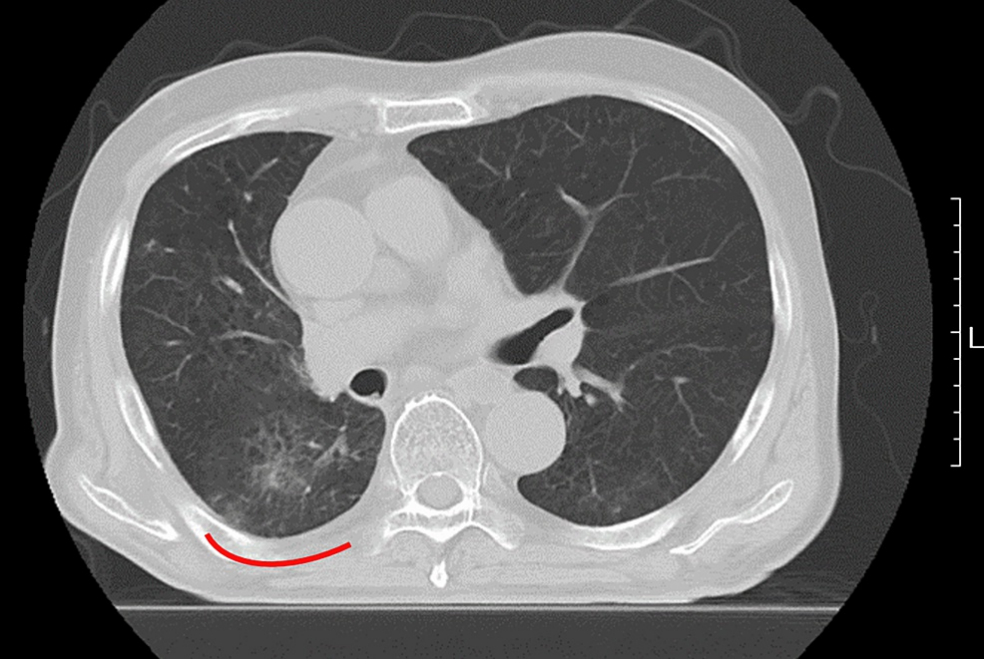
Diagnostic CT image on the same day RP was suggested by the CBCT images RP is indicated with a red line. CBCT - cone-beam computed tomography; RP - radiation pneumonitis

**Figure 7:**
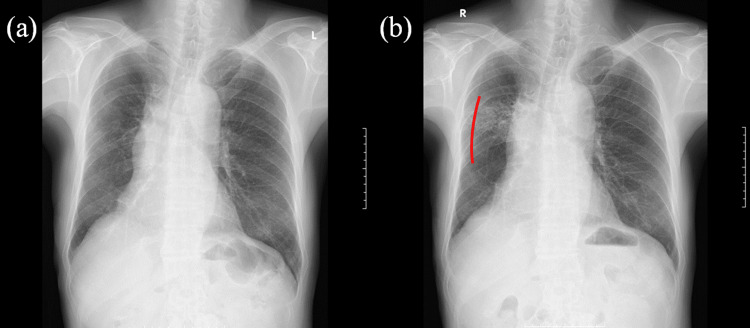
X-ray photographs X-ray photograph (a) on the day the CBCT image suggested RP at a cumulative dose of 50 Gy, and X-ray (b) three weeks later showing RP indicated with a red line. Radiotherapy was continued to a full dose of 60 Gy due to a strong request from the patient, with oral prednisolone administration. CBCT - cone-beam computed tomography; RP - radiation pneumonitis

CT scan revealed primary lung cancer with spinal metastases. Figure 4 shows a treatment plan, and Figures 5a-b indicate CBCT images on the same day RP was suggested. Figure 6 represents a diagnostic CT image acquired on the same day. Figures 7a-b compare X-ray photographs on the day RP was suggested by CBCT and three weeks later. In this case, RP was found on the CBCT images at 50 Gy, but the radiotherapy was continued to a full dose of 60 Gy due to a strong request from the patient. The patient had mild inflammatory symptoms with KL-6 serum level of 903 U/mL at 56 Gy. Dose deliveries from 52 Gy up to 60 Gy were performed under the very careful observation of daily CBCT images. RP was successfully cured after 4.5-month outpatient administration with oral prednisolone. The patient survived for five years without recurrence.

Case 3

**Figure 8:**
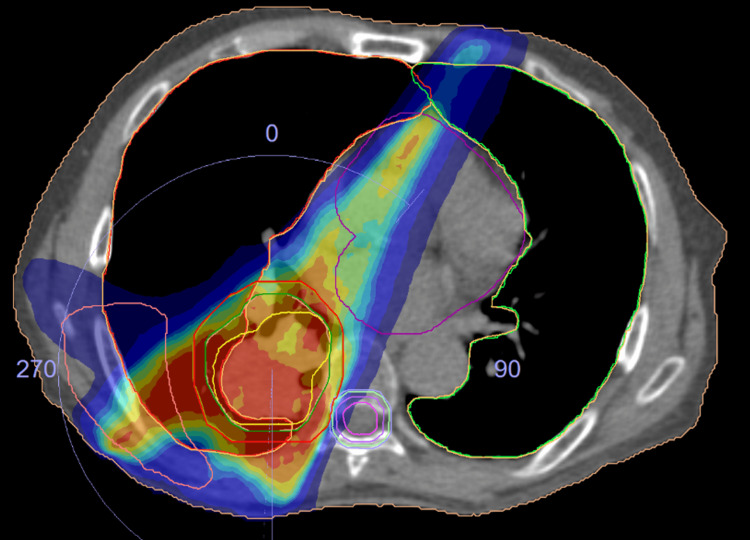
A treatment planning result with dose color wash ranging from 20 Gy (dark blue) to 60 Gy (dark orange) CTVs in the maximum exhalation and the maximum inhalation phases were combined to define an ITV in green, and then PTV (dark orange) was defined by adding a 5 mm margin to the ITV, and finally, a spinal PRV + 2 mm region (sky-blue) was excluded. CTV - clinical target volume; ITV - internal target volume; PTV - planning target volume; PRV - planning organ at risk volume

**Figure 9:**
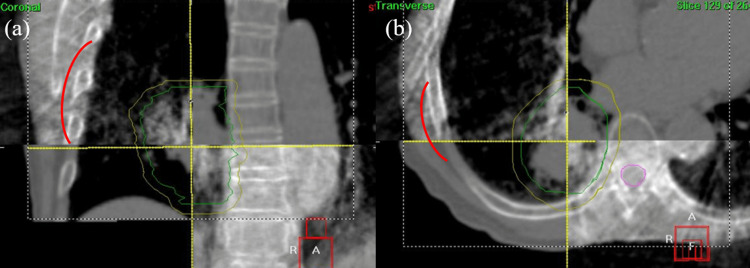
Four-quadrant matrix display of CBCT (upper left and lower right) and planning CT (lower left and upper right) images in (a) coronal and (b) axial planes CBCT images were acquired with a bone window setting, and increased reticular shadows were observed at the right of the tumor, as shown with the red lines, which suggests possible RP. Green and yellow lines show the CTV and PTV contours, respectively. CBCT - cone-beam computed tomography; RP - radiation pneumonitis; CTV - clinical target volume; PTV - planning target volume

**Figure 10:**
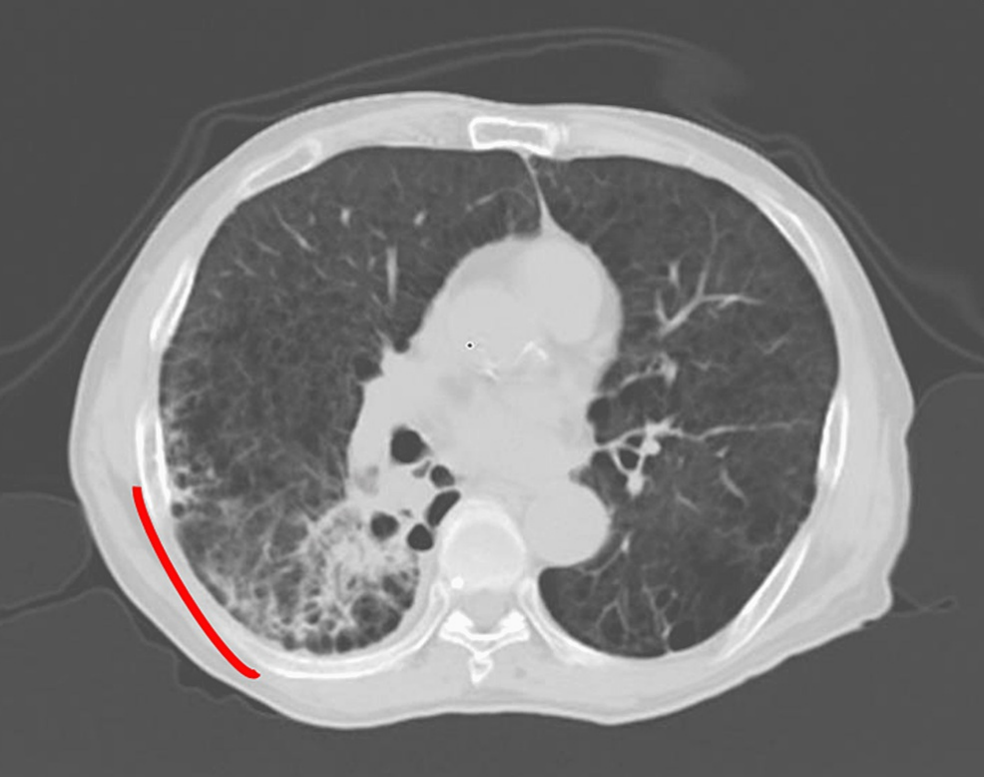
Diagnostic CT image on the same day RP was suggested by the CBCT images RP is shown with a red line. RP - radiation pneumonitis; CBCT - cone-beam computed tomography

**Figure 11:**
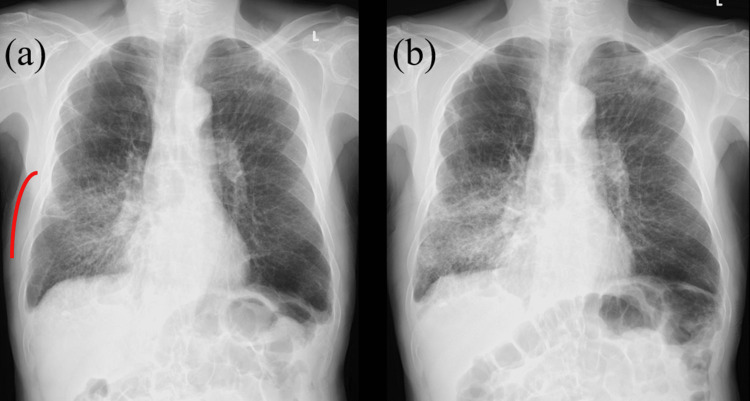
X-ray photographs acquired (a) two weeks (RP marked by a red line) and (b) three weeks after terminating radiotherapy, the latter showing more significant RP RP - radiation pneumonitis

This patient, who had pre-existing obstructive pneumonia and slight pretreatment pulmonary fibrosis with a score of 1 [5], experienced severe grade 3 RP. The diffusing capacity of the lungs for carbon monoxide (DLCO) was examined prior to the radiotherapy, which was reduced to 49%. Radiotherapy was terminated at 58 Gy, but CBCT had suggested possible GGO earlier, even with a bone window setting. KL-6 levels prior to radiotherapy and at the time of termination were 470 U/mL (slightly higher than the normal range) and 630 U/mL, respectively. After one month of intravenous pulse prednisolone administration, the KL-6 level was stabilized to 670 U/mL, leading to relief and cure. Because the initial blood test showed leukocytosis, we could not diagnose the patient with RP earlier, which delayed prednisolone administration. Figure 8 shows a treatment planning result, and Figures 9a-b represent a four-quadrant matrix display of CBCT and planning CT images on the day RP was suggested. Figure 10 indicates a diagnostic CT image on the same day RP was suggested by the CBCT. Figures 11a-b compare X-ray photographs acquired on different days after terminating the radiotherapy.

Case 6

**Figure 12:**
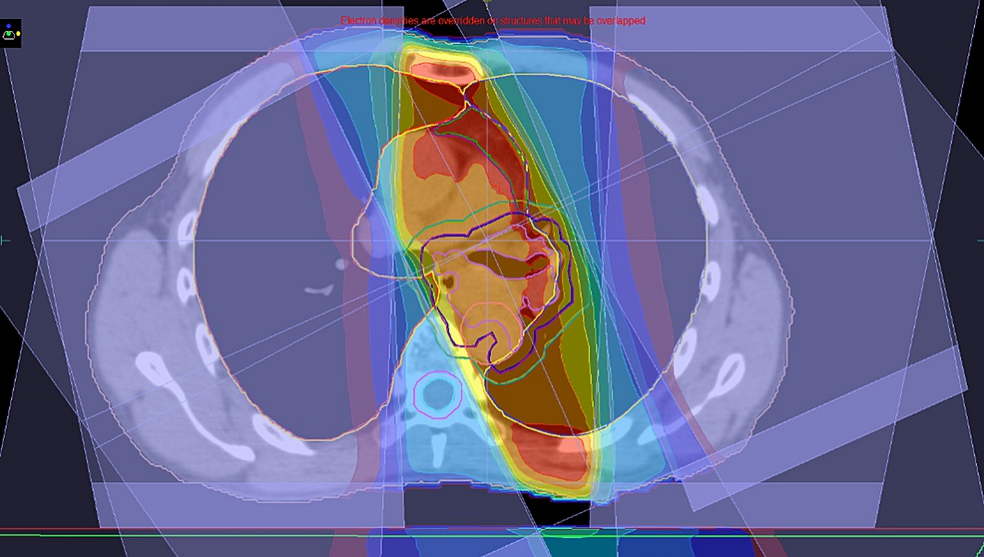
A treatment planning result with structures and dose color wash CTV and PTV contours are shown with purple and green colors, respectively. Left mediastinal lymph nodes were also electively treated, where CTV and PTV are indicated with the same colors as above but overlapped because a treatment margin of 0 mm was employed. CTV - clinical target volume; PTV - planning target volume

**Figure 13:**
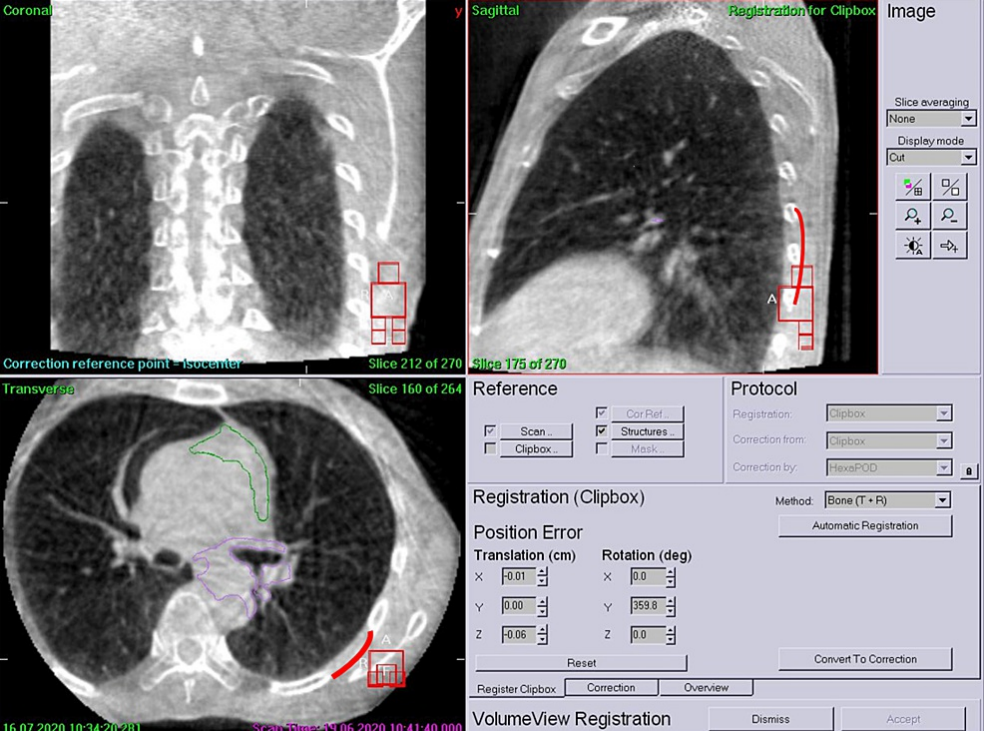
CBCT images displayed in three orthogonal planes At a cumulative dose of 43.2 Gy, CBCT suggested GGO on a sagittal plane posteriorly, as shown by red lines. Purple and green lines on the axial plane show the CTV contours of the primary tumors and the elective lymph nodes, respectively. CBCT - cone-beam computed tomography; GGO - ground-glass opacity; CTV - clinical target volume

**Figure 14:**
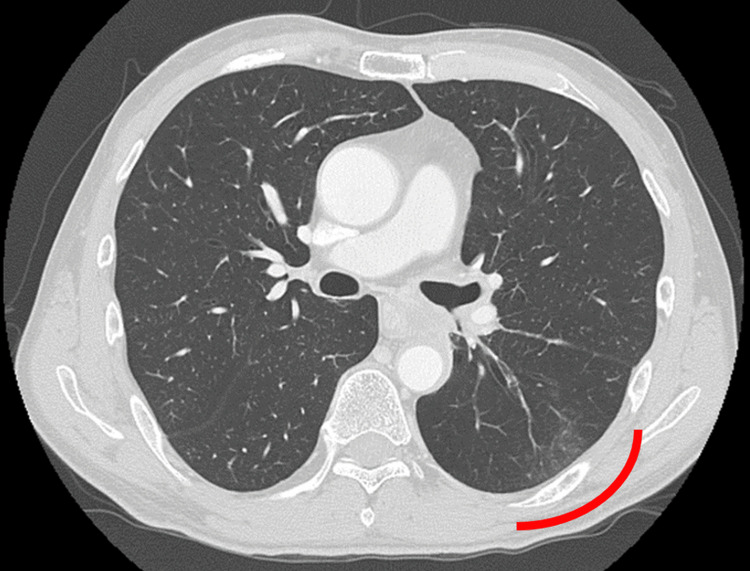
CT image on the same day RP was suggested by the CBCT images, where GGO was confirmed at the left lung S6, as shown with the red line RP - radiation pneumonitis; CBCT - cone-beam computed tomography; GGO - ground glass opacity

CT scan revealed central lung cancer with enlarged hilar lymph nodes. Figure 12 shows a treatment planning result, and Figure 13 indicates CBCT images that suggested GGO as the red lines. Figure 14 shows a diagnostic CT image acquired on the same day RP was suggested by the CBCT.

**Figure 15:**
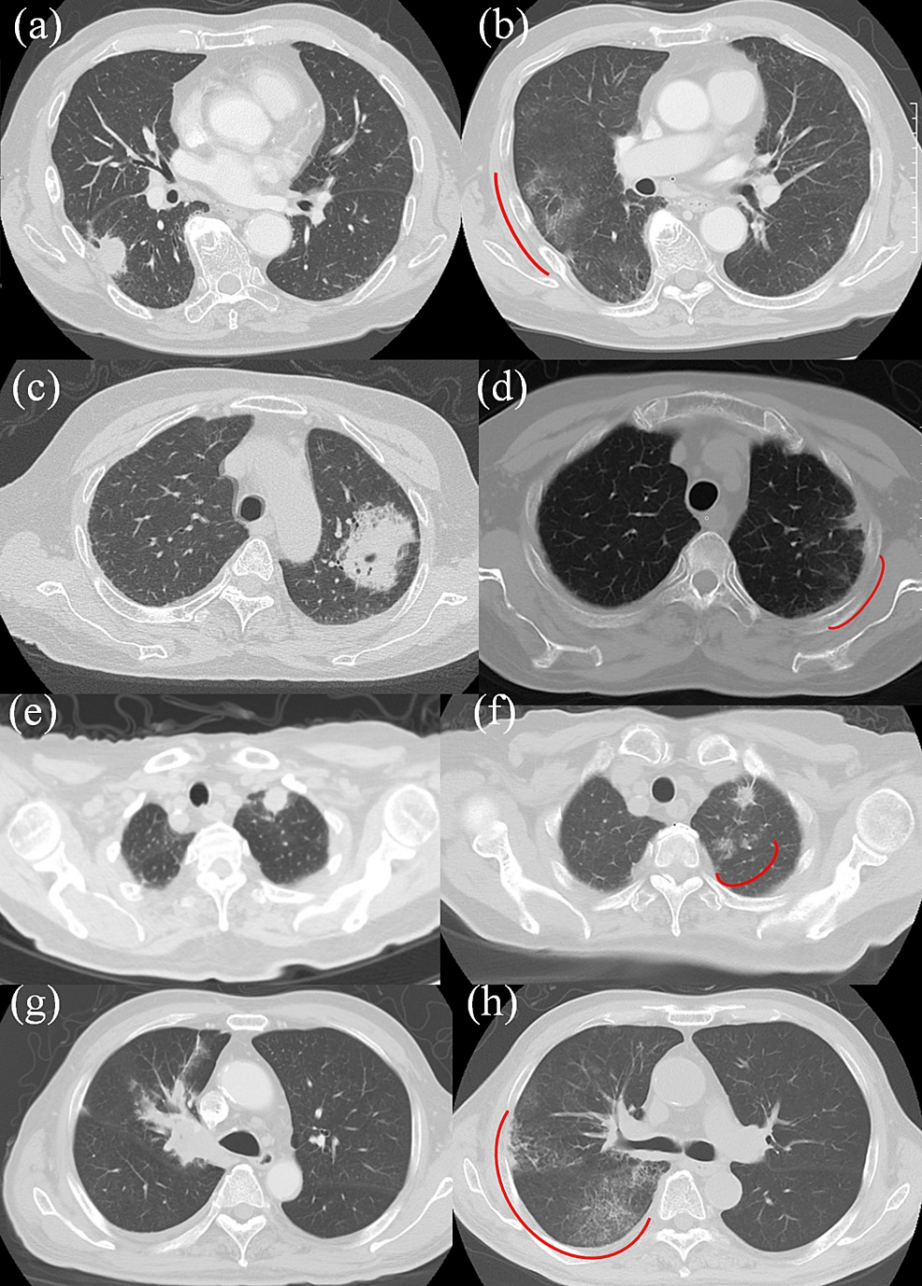
Comparison of CT images at the times of treatment planning and RP detection in cases 4 (a, b), 5 (c, d), 7 (e, f), and 8 (g, h), respectively RPs are indicated with red lines. RP - radiation pneumonitis

For the remaining cases, 4, 5, 7, and 8, CT images at the times of treatment planning and RP detection are shown in Figures 15a-h, respectively. Case 4 resulted in the most severe grade 3 RP among all the eight cases, with KL-6 level of 1200 U/mL on the day the radiotherapy was terminated and then reached 3950 U/mL after three weeks of prednisolone oral administration. Further administration of two-week intravenous pulse prednisolone followed by high-dose oral prednisolone finally reduced the KL-6 level to 1800 U/mL, leading to relief and cure. Case 4 received chemotherapy prior to radiotherapy, which may be a cause of the grade 3 RP. As mentioned earlier, cases 5 and 7 resulted in asymptomatic grade 2 RP, each being confirmed by increased levels of KL-6 and CRP. The pretreatment pulmonary fibrosis was barely observed in cases 4 and 5 but with the same score of 1 as in case 3. Cases 7 and 8 have relatively low V20 of 14% and 15%, respectively, both leading to grade 2 RP. Of note, local recurrence was observed only in case 1 (after five months).

As mentioned earlier, 22 patients out of the 161 patients were diagnosed with RP after completion of their radiotherapy courses. Table 2 shows RP grades, months to develop RP after completion of radiotherapy, pretreatment pulmonary fibrosis status and its score, and pre- or concurrent chemotherapy status of these 22 cases. It was found that nine cases had pretreatment pulmonary fibrosis with a score of 1, which seems very frequent. Of further note, a patient with an estimated V20 of around 32 to 34% was diagnosed with grade 5 one month following the radiotherapy. Because the patient refused hospitalization, RP management was delayed. In addition, higher V20 increases higher grade RP risks [1]. 

**Table 2:** The characteristics of 22 patients who were diagnosed with RP following their radiotherapy courses This table comprises RP grades, months to develop RP after completion of radiotherapy, pretreatment pulmonary fibrosis status with its score, pretreatment COPD stages, and pre- or concurrent chemotherapy status. Stage 0 of COPD means that assessed COPD was negative, whereas "-" shows no COPD assessment. + slightly; ± barely; * gefitinib; ** durvalumab RP - radiation pneumonitis; d - days; COPD - chronic obstructive pulmonary disease

RP grade	1								2					3								5
Months to RP	0.5	1	1	1	1.5	2	3	4	2d	20d	2	2	3	7d	1	1	1.5	1.5	2	4	12	1
Pretreatment pulmonary fibrosis	-	±	±	-	-	-	±	-	±	-	±	-	-	±	±	-	-	-	-	-	±	+
Score	0	1	1	0	0	0	1	0	1	0	1	0	0	1	1	0	0	0	0	0	1	1
Pretreatment COPD stages	-	-	-	II	0	0	-	-	I	-	0	0	0	-	I	-	0	IV	0	-	III	0
Pre- or concurrent chemotherapy	-	-	-*	-	+	-	-	-	+	-	+**	-	-	-	-	+	-	-	+	-	-	-

## Discussion

We have been making the best efforts in detecting early GGO and early RP on CBCT images prior to symptoms. Initially, RP was detected at 50 Gy or greater. However, more careful CBCT observation resulted in earlier detection at around 40 Gy. Then, a new problem has arisen whether the treatment should be terminated. Sanuki et al. [7] reported that early RP development was associated with higher-grade complications, and therefore it is preferable to terminate radiotherapy once we detect even early-phase RP. However, termination in the middle of the treatment course may significantly reduce the therapeutic effect. In our experience, patients with favorable clinical status may continue to receive radiotherapy with careful observation of lung parenchyma on CBCT images and clinical data such as KL-6 and CRP.

As was also described, the RP lesions found on the CBCT images in the eight cases were all cured after administering oral prednisolone (PSL) to grade 2 patients and intravenous pulse PSL to grade 3 patients. This may be the largest advantage of our early RP management. Tsujino et al. [6] reported that pretreatment pulmonary fibrosis led to higher-grade RP. In this study, three out of eight cases had pretreatment pulmonary fibrosis, and two of them resulted in grade 3 RP.

We have recently experienced two cases of very localized radiation to severe esophageal stenosis with lymph node recurrence under careful monitoring of RP through daily CBCT scan. CBCT-based RP monitoring resulted in satisfying treatment outcomes even with pretreatment pulmonary fibrosis of score 2 [6]. Once we find RP on CBCT at the early stage of the radiotherapy course, a decision of termination or continuation is made depending on the patient's clinical condition. When early GGO signs are found on CBCT, our institutional decision of termination is made after delivering an additional dose of 2 to 7 Gy to confirm definite RP. This is based on our experience that early localized GGO would not immediately lead to a lethal consequence. In our hospital, 42 Gy is the minimum dose for radiotherapy termination when early phase RP is confirmed during the treatment, and in six cases out of eight, the treatment sessions were terminated after delivering 50 Gy. Our clinical practice is that CBCT images are carefully observed after reaching 40 Gy and the most intensively monitored after 50 Gy. More specifically, after reaching 50 Gy, we monitor in-field lung tissues following each beam delivery by changing the CBCT window setting from bone to lung to check if GGO or RP may arise.

Limitations

This is our observational study with a limited number of cases. More clinical data would be required in larger institutions to establish a solid treatment strategy for early RP detection. Recently, Jose et al. [15] proposed a machine learning algorithm for early RP detection using CBCT image-based delta radiomics. In the future, this technique may provide an automated method for detecting early RP more precisely during the course of radiotherapy.

## Conclusions

We have shown that early detection of RP may be feasible during radiotherapy courses by daily monitoring of CBCT lung images. The sample size is small and limits the extent to which the findings in this study can be generalized. Further studies with larger data sets are awaited to proceed.
